# The silent invader: a case of intrapericardial hydatid cyst with exceptional pulmonary artery involvement

**DOI:** 10.1093/omcr/omad099

**Published:** 2023-09-25

**Authors:** Aida Soufiani, Samah El-Mhadi, Hamza Chraibi, Zineb Agoumy, Zineb Fassi Fehri, Sanae Es-sebbani, Hasnaa Leghlimi, Omar Ech-cherif El Kettani, Fadoua Lachhab, Mohammed Tribak, Rokya Fellat, Nesma Bendagha, Said Moughil

**Affiliations:** Cardiovascular Surgery B Department, Ibn Sina University Hospital, Mohammed V University, Rabat, Morocco; Cardiology A Department, Ibn Sina University Hospital, Mohammed V University, Rabat, Morocco; Cardiology A Department, Ibn Sina University Hospital, Mohammed V University, Rabat, Morocco; Cardiology A Department, Ibn Sina University Hospital, Mohammed V University, Rabat, Morocco; Cardiology A Department, Ibn Sina University Hospital, Mohammed V University, Rabat, Morocco; Cardiology A Department, Ibn Sina University Hospital, Mohammed V University, Rabat, Morocco; Cardiovascular Surgery B Department, Ibn Sina University Hospital, Mohammed V University, Rabat, Morocco; Cardiovascular Surgery B Department, Ibn Sina University Hospital, Mohammed V University, Rabat, Morocco; Cardiovascular Surgery B Department, Ibn Sina University Hospital, Mohammed V University, Rabat, Morocco; Cardiovascular Surgery B Department, Ibn Sina University Hospital, Mohammed V University, Rabat, Morocco; Cardiology A Department, Ibn Sina University Hospital, Mohammed V University, Rabat, Morocco; Cardiology A Department, Ibn Sina University Hospital, Mohammed V University, Rabat, Morocco; Cardiovascular Surgery B Department, Ibn Sina University Hospital, Mohammed V University, Rabat, Morocco

## Abstract

A 70-year-old woman was referred to our cardiology department for the management of dyspnoea. Cardiovascular examination revealed a loud P2, with no sign of right-sided heart failure. Chest X-ray showed a convex left medium cardiac border and a double contour along the right cardiac border. Transthoracic echocardiogram revealed a cystic mass attached to the right ventricle apex. Computed tomography scan showed cyst with fluid density on the apex of the right ventricle; and a honeycomb-like aspect cyst with partial occlusion in the left pulmonary artery. Cardiac magnetic resonance imaging revealed the presence of hydatic intrapericardial cyst that compresses the right ventricular apex; associated with intraluminal left pulmonary artery cyst. Hydatic serology was positive. The patient refused surgery and was discharged on a regimen of Albendazole. She has been followed up closely with a good outcome.

## INTRODUCTION

Hydatidosis is a systemic zoonosis caused by the larval stages of the cestode *Echinococcus granulosus*, which can occur in any organ of the body via the general or lymphatic circulation. Cardiac involvement in hydatidosis is uncommon.

## CASE REPORT

A 70-year-old female patient was referred to our cardiology department for the management of chronic dyspnoea with a New York Heart Association functional class of III. She had no familial or personal medical history.

Upon admission, the patient had normal blood pressure and heart rate. She was not tachypneic and had normal peripheral oxygen saturation on ambient air. Cardiovascular examination revealed a loud P2 with no pulmonary crackles nor signs of right-sided heart failure.

Electrocardiogram revealed T-waves inversion in inferior and anterior leads with no features of right heart strain.

Postero-anterior chest X-ray showed a leftward convex medial cardiac border, a double contour along the right cardiac border and multiple opacities of different size.

**Figure 1:**
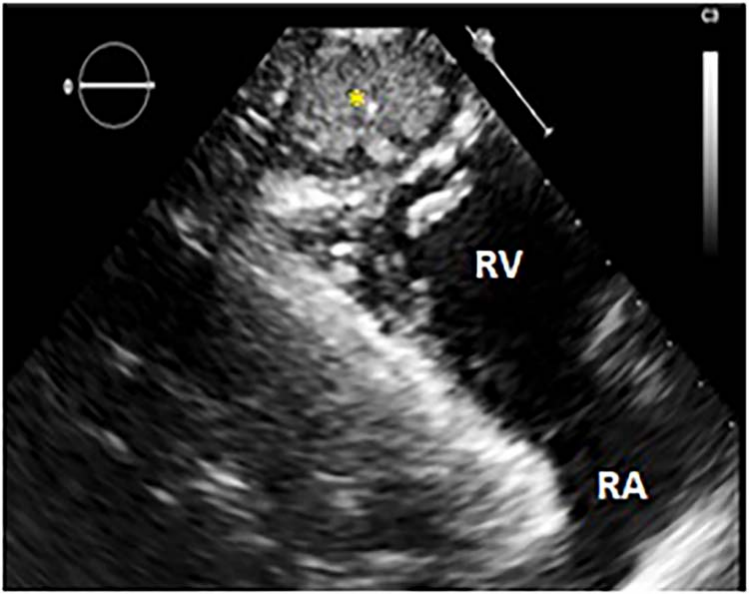
TTE: modified parasternal long axis view showing a hyperechoic cystic mass (yellow star) next to the right ventricular apex. RV: right ventricle; RA: right atrium.

Echocardiography (TTE) was performed and showed a cystic mass measuring 23*38 mm with echo-negative content, attached to the right ventricular apex (Fig. 1). Parasternal short axis view at the level of the aortic valve showed dilation of the pulmonary artery’s trunk, and high probability of pulmonary hypertension based on the combination of a systolic peak tricuspid regurgitation velocity at 3.34 m/s, pulmonary artery dilation (pulmonary artery diameter > aortic root diameter) and TAPSE/sPAP ratio at 0.42 mm/mmHg. Heart valves, atria, left and right ventricular function were normal.

**Figure 2:**
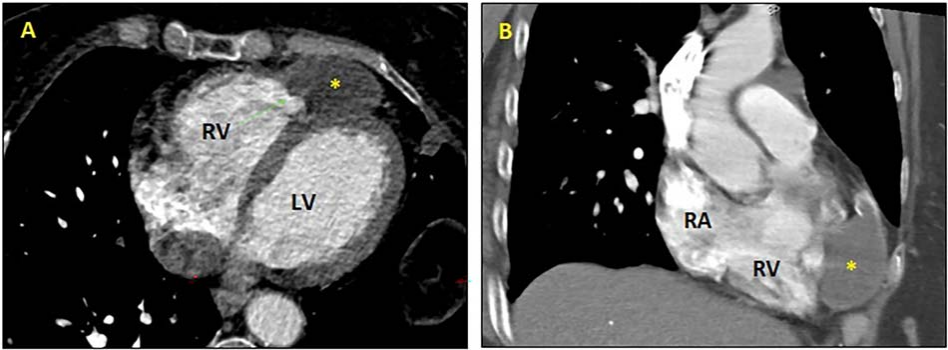
CT scan: axial (**A**) and sagittal (**B**) views showing hydatic intrapericardial cyst (yellow star) on the right ventricle’s apex. RA: right atrium; RV: right ventricle; LV: left ventricle.

**Figure 3:**
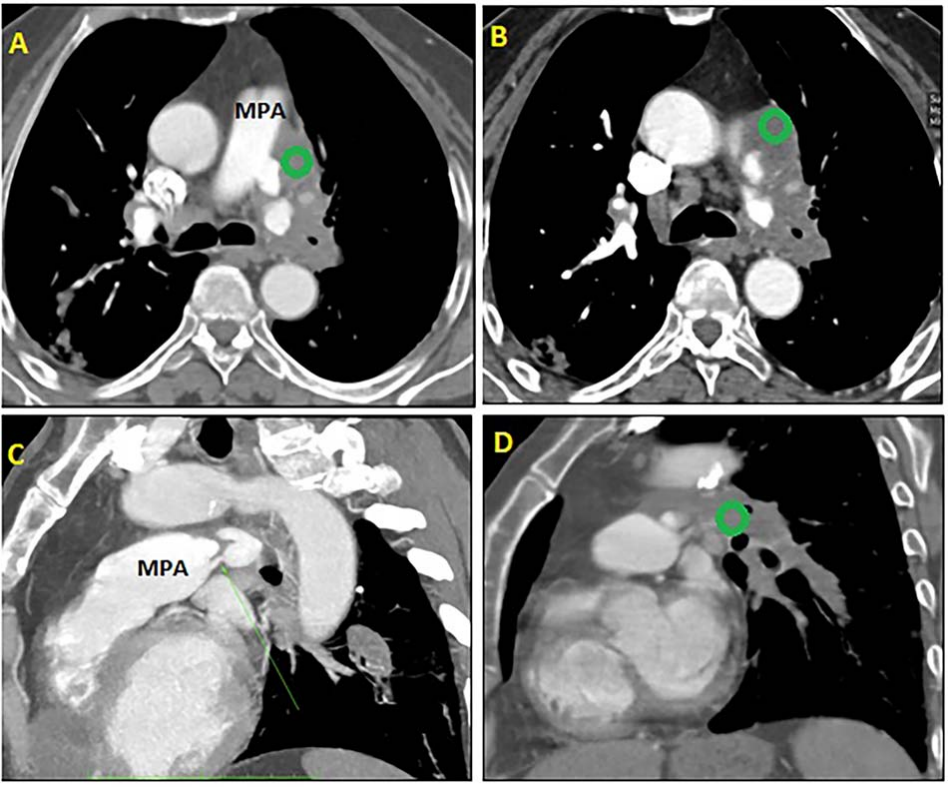
CT scan: axial (A + B) and sagittal (C + D) views showing a multivesicular hydatic cyst (green circle) class CE2 WHO, with severe reduction of the flow and partial occlusion of the left pulmonary artery. MPA: main pulmonary artery.

Thoracoabdominal computed tomography (CT) scan showed cyst with fluid density on the apex of the right ventricle (Fig. 2); and a multivesicular, multiseptated, honeycomb-like cyst [class CE2 World Health Organization (WHO)] with partial occlusion in the left pulmonary artery (Fig. 3). The liver was free of cysts.

**Figure 4:**
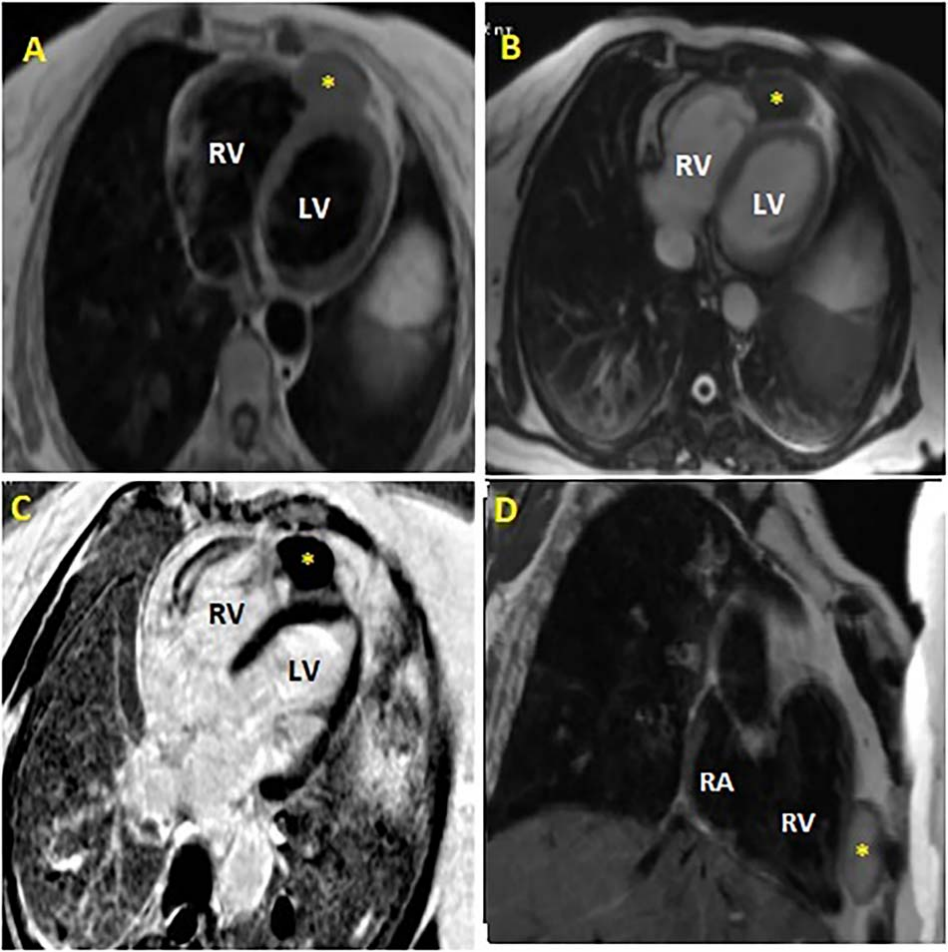
Cardiac magnetic resonance imaging (MRI) showing hydatic intrapericardial cyst (yellow scar) involving the myocardium at the right ventricular apex, with intermediate signal on T2-weighted imaging (**A**: axial view, **D**: sagittal view), iso-intense to myocardium on T1-weighted imaging (**B**: axial view) and hypo-intense to myocardium on late gadolinium enhancement (LGE) axial view (**C**). RA: right atrium; RV: right ventricle; LV: left ventricle.

**Figure 5:**
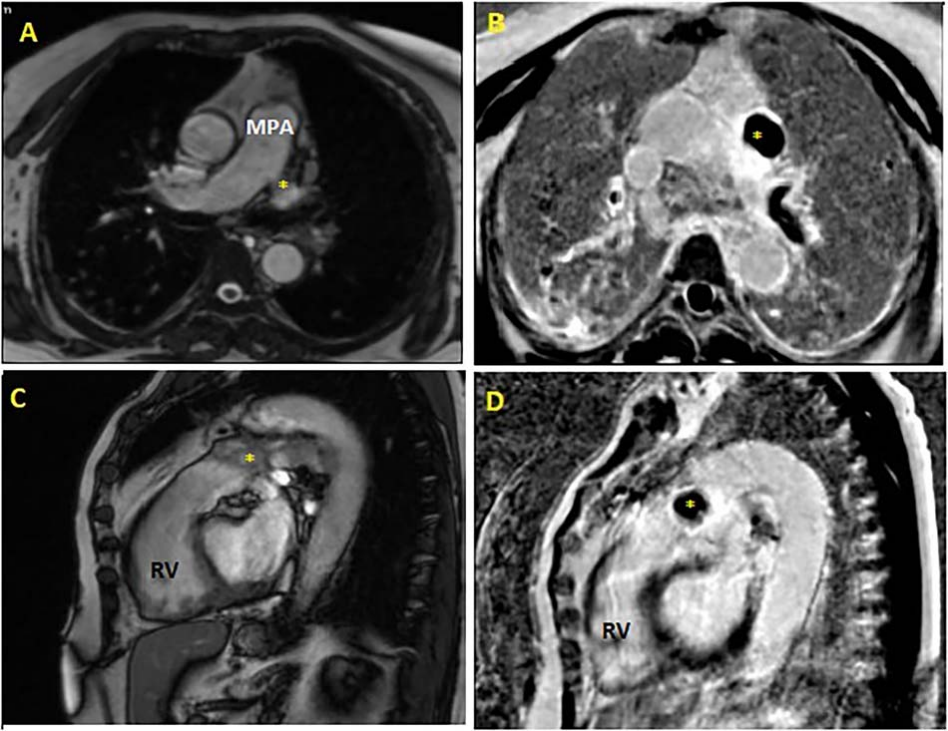
Cardiac MRI: cine axial view (**A**) and cine sagittal view (**C**) revealing intraluminal active multivesicular honeycomb-like cyst in the left pulmonary artery, hypo-intense in LGE sequences (axial view: **B**, sagittal view: **D**). MPA: main pulmonary artery; RV: right ventricle.

The patient was referred to cardiac magnetic resonance imaging (CMR) unit for further investigation. CMR revealed the presence of hydatic intrapericardial cystic with partially calcified wall that compresses the right ventricular apex (Fig. 4), associated with intraluminal left pulmonary artery cyst measuring 12 × 8 mm (Fig. 5). Both of them had hypointense T1-weighted and hyperintense T2-weighted image.

Dot immunogold filtration assay was performed and hydatid antibodies were detected.

**Figure 6:**
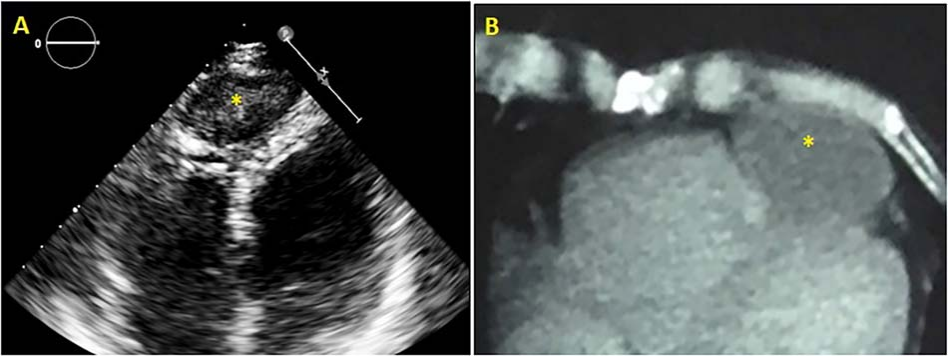
TTE apical four chamber view (**A**) and CT scan axial view (**B**) showing intrapericardial hydatid cyst’s evolution to the class CE4/5 (WHO) 2 years after discharge.

Since the patient refused surgery, she was discharged home on a regimen of Albendazole, 15 mg/kg daily for four cycles (4-weeks cycle followed by a 2-week albendazole-free interval). The patient has been followed up closely, and showed progression to an inactive form of cysts (class CE4 WHO) with exclusive anthelminthic therapy (Fig. 6).

## DISCUSSION

Hydatidosis refers to parasitic infection induced by the larvae of *E. granulosus.* Hydatid cyst is still endemic in developing countries, particularly among sheep breeders, and is considered as a public health problem in Morocco [1].

Humans can be infested directly via close contact with contaminated dogs or indirectly after ingesting water or food containing tapeworm eggs. After ingestion, the oncosphere crosses the duodenal mucosa, penetrates the venous and/or lymphatic system, and may affect any part of the body. The liver (70%) and lungs (25%) are the most affected [2].

Cardiac involvement is seen only in 0.2-3% of all cases. This rareness is due to the continuous contraction of the heart that prevents the attachment of parasite eggs into the cardiac wall [3].

In primary cardiac hydatidosis, larvae usually reach the myocardium through the coronary circulation, via the pulmonary circulation or a patent foramen ovale; although the intestinal lymphatic vessels, the thoracic duct, the superior and inferior vena cava, the haemorrhoidal and the pulmonary veins may be involved [4].

Multiple sites of cardiac hydatidosis may occur in a single patient, and the most commonly affected ones are the left ventricle (75%) and right ventricle (18%). Pericardium (10%), left atrium (8%), right atrium (4%) and interventricular septum (4%) are less frequent locations. Pulmonary artery hydatid cysts are exceptional and are generally the consequence of embolisms from primary cardiac locations [5].

Cardiac Echinococcosis is frequently asymptomatic (90%). In the other 10% of cases, symptoms are non-specific and depend on the number, the size, the evolutionary stage, the location and the local damage of the cysts.

Serological tests are important and have the ability to confirm the diagnosis of hydatidosis when the results are positive. The identification of cardiac cyst lesion (TTE and/or CT scan and/or CMR) associated with positive serology makes the diagnosis [6].

Echocardiography detects cysts with good sensitivity except for particular locations, so the left pulmonary artery cyst has not been diagnosed in our patient case. The presence of multivesicular appearance or membrane detachment is highly suggestive of the hydatid origin of cysts [7].

Echocardiography also helps to assess the stage of hydatid cysts according to the classification proposed by the WHO in 2001 and plays a crucial role in determining the appropriate management based on the cyst’s stage [8].

CT is useful to corroborate the diagnosis and check the extension and anatomic relationships of the cyst. In case of doubt or if there is a discrepancy between TTE and CT scan, CMR can be performed. The cyst presents as an oval lesion with hypo intense and hyper intense signals on T1-and T2-weighted sequences, respectively [8].

Nowadays, surgical cystopericystectomy is still the gold standard of cardiac hydatidosis management. In order to prevent perioperative dissemination and to reduce postoperative recurrences, resection under cardiopulmonary bypass combined to the use of albendazole therapy is the safest approach [9].

The prognosis of cardiac hydatidosis is poor because of the risk of acute fatal complications such as obstruction of the ventricular outflow chambers, valvular dysfunction, compression of the conduction pathways and/or coronary arteries, pulmonary embolism and anaphylactic shock [10]. Right ventricular location is quite dangerous. Hydatic heart cysts have a tendency to rupture and cause pulmonary embolism, as for our patient. Acute pulmonary embolism should be kept in mind in patients who have right ventricle hydatidosis if suddenly chest pain and/or dyspnoea occur.

The therapeutic management is difficult and often partially effective; hence the importance of focusing on preventative treatment. Prevention of hydatidosis consists on treating dogs that may carry the disease and encouraging sheep vaccination.
